# Address Delivered before the Society of Alumni of the Baltimore College of Dental Surgery

**Published:** 1877-04

**Authors:** W. W. H. Thackston

**Affiliations:** Virginia.


AMERICAN JOURNAL

OF

DENTAL SCIENCE.

Vol. X. THIRD SERIES—APRIL, 1877. No. 12.

### ARTICLE I

*Address delivered before the Society of Alumni, of the Baltimore College of Dental Surgery, March, 9th, 1877.*

BY W. W. H. THACKSTON, M. D., D. D. S., OF VIRGINIA.

MR. PRESIDENT, FELLOW MEMBERS OF THE SOCIETY OF ALUMNI.—In the name of the College whose training you have enjoyed, and whose honors you wear, I salute you, in the names of the illustrious dead who gave you Alma Mater, and whose spirits may now be hovering benignantly over this presence and this occasion; I give you the "all hail" of an elder brother, whose eyes beheld the dawn of that era that lifted your chosen calling to the dignity of a science, and to the place of an honored and acknowledged profession.

The circumstances surrounding us, this splendid building, these spacious halls, garnished and equipped with all the appliances of progress and improvement, the books, maps and charts, the museum, the infirmary, dispensary and laboratory; with a corps of teachers representing every department and subdivision of dental science, and this presence, this crowd of Alumni, fill us with emotions we need language to express.

When we recall the circumstances and incidents of our personal connection with this first, and pioneer school, when it stood solitary and alone of its kind in the world—when four true and brave men composed its faculty—when a modest little room, in a comparatively quiet and obscure street, accomodated its classes, when five graduates comprised its Alumni and were the sole representatives of its work—when the responsibility of success or failure in a great enterprise, destined to affect the health and happiness of unnumbered millions of the human family rested upon *Four* teachers, and *Five* accredited students; and when we remember that little more than three decades have intervened, and now contemplate the spectacle before us; we are filled with unutterable wonder and amazement, and feel more than ever impressed by the conviction, that the work of our *Fathers* was a true and genuine inspiration. And here near by the lasting resting places of these great and true men, upon the ground which they consecrated—before an assembly, such as they only beheld through the visions of a sublime and heroic faith, we realize their mysterious presence, we again hear their encouraging voices, and catch some beams of the light that hallows their immortal brows, in the "Land beyond."

We feel, Mr. President and Fellow Alumni, that in this hour and in this presence, the most acceptable offering we can bring to the memory and characters of Harris, Hayden, Handy and Bond; and the most valuable service we can render you and the calling you honor, will be a vindication of the position assumed and maintained, and the policy inaugurated and illustrated in the lives and labors of these distinguished founders of our profession. And in doing this, we shall endeavor to fix the landmarks, and trace the pathway for your guidance as faithful Alumni, as filial and loyal Sons of an honored mother.

We are conscious, that in this utilitarian age and day, when all objects and results are measured and estimated according to their money value; when society is engrossed and absorbed in the solution of the *paying* problems of life, that sentiment—however elevating and ennobling, is at a discount. Men wrestling in the struggle for existence, for wealth and for position, reck little of the finer feelings and purer elements of human character, the chivalrous instincts and general impulses of our nature are held in obeyance, or stifled and stilled by our grosser passions and grovelling pursuits. Our ends and aims are for the most part selfish, and our means and methods often vulgar and soulless.

But happily for us all, there are incidents and occasions scattered along the pathway of life, that loose, and give play to the heartstrings, and bring us back to the exercise and enjoyment of those attributes and qualities that most refine and exalt our moral natures; and of such may we not claim the present?

The love of Alma Mater—all else to the contrary—is honorable and elevating; the '*esprit de corps*' which links together the accredited students of our honored school as a band of brothers, as co workers and co laborers in the cultivation and practice of a humane and benevolent profession, is no less elevating and ennobling in its *individual* effect and influences, than potent and powerful in its general operation upon the truest and highest professional development. It is untrue, that love of Alma Mater is a barren and sickly sentiment, it is a healthy and vital principle, that refines and adorns individual character—that incites to generous and active effort—that nurtures true progress, and yields a rich fruitage to the harvest of human knowledge and human power. We applaud the exercise and the illustration of the fraternal sentiments and feelings, and the exhibition of that filial devotion that attracts and brings together the widely separated children of an honored mother, the Alumni of a noble school on occasions of social recreation and enjoyment, and in transactions of general professional interest and advantage.

But Mr. President, the body over which you have the distinguished honor to preside, is not a mere social or festive appendage of Alma Mater; its design is use, rather than ornament, "*labor* as well as refreshment," and to honor ourselves, as well as the usage of our Masonic brethren, we should heed the call to the first, with as much alacrity as to the last. We must *work*, and then our recreation and refreshment will be spiced and seasoned with the conscious performance of duty, with the approving sense of fidelity to our trusts, and then may we claim from our Foster Mother the plaudit, "well done good and faithful Sons."

What organization can be more competent to determine the demands of society upon our profession than a society of Alumni? what body could more wisely, or more justly estimate the value and resources of our school? and who more capable of a clear apprehension, and wise discrimination in all that pertains to our educational interests and professional needs than the members of this Alumni Association?

As we have intimated, we propose to advocate the system of separate and distinct schools, as best adapted to the educational necessities of our profession, and to present the "Baltimore College of Dental Surgery," as a model and exemplar of this policy—as the first, the oldest, the most successful, and deservedly the most honored of all the schools which have been founded upon the system it originated and established, and which it has so brilliantly illustrated in its past history.

But why, we may be asked, select such a subject for this occasion? Has not the Old Baltimore College demonstrated the proposition, that a special school for a special division of science is more attractive, more efficient, and more successful, at least in our deportment and domain, than any system or device of the past or any method or arrangement of this present marvelous age of transition and progress? Has not the Baltimore College and the agencies and influences it originated and put in operation, absolutely created our profession? Has not the principle and system upon which this first of all dental schools was founded, been recognized and adopted in every section of our common country, and almost all over the civilized world, and always and everywhere, with a more or less gratifying and compensating success? The great commercial Cities of the North, the East, the West, and the South, have their special schools for teaching dental science—some of the Universities have added faculties of instruction, and established departments for teaching dental surgery—all essentially modeled after the first grand and original design, conceived by the Fathers of American Dentistry, who were once, the honored members of this community—who lived, and labored, and died in this great City—and whose names will live, and whose memory will be cherished, when the princely pallaces and proud monuments that tower above us shall have crumbled into dust. What need of discussing such a theme, before such an audience as we have to-day?

Mr. President, the fact is potent, and has not escaped your observation, that the present is an age and day of feverish impulse, and restless dissatisfaction, of eager desire for change, and what is called progress in all the pursuits of life; and to a given extent, it is perhaps well that it is so. We would not stand still and rust or stagnate, nor would we forever run in the same old grooves and channels; but great as are the results of activity and progress—the safety of society, and the interests of every calling and pursuit, demand that the "brakes" should sometimes be applied,—the sails reefed and furled, and the anchor dropped.

In our wild enthusiasm, our desire for change and progress, our judgments become unbalanced, we lose our mental and moral polarity; and in our ill-considered and erratic efforts to accomplish some startling result, some mighty change, and some great advance; we let go the good, the true, and the tried, and catch at the illusory, the barren, and the worthless.

Sirs, in the face of the most wonderful achievements which any professional history records; in the presence of a growth and development which staggers human belief, and almost appals human credulity; in the presence of a profession created by this grand old school, by its wise adjustment of means to ends—and lifted in less than half a century to a position of respectability and commanding importance all over the civilized world—the cry is raised, that special schools and colleges for our department of science are inadequate to the present demands of society, and of our profession; that we should give up and abandon colleges of Dental Surgery, and should seek a higher plane, and broader and more thorough culture, through the medium and agency of Medical Schools, and a more dignified and respectable title to public confidence and esteem than our distinctive College Degree. Strange to say, these preposterous and suicidal ideas, have not only found a lodgment in the heads of some of our most worthy and influential dentists, but have been caught up and adopted as a rallying cry with some of our professional organizations—and at least one of our popular periodicals, has not hesitated to express approval of the design to relegate the profession of Dental Surgery to the care and control of the purely medical institutions of the country. These ideas and movements demand our recognition, nay more, demand an exposure of their folly and absurdity, and an expression of disapproval and condemnation, that will forever fix the seal of doom upon them.

Forgetful of the teachings of a sad history, and a bitter, and more sad and humiliating experience, running down through a whole century preceding the estabiishment of this College, it is now alleged, "that as Dental Surgery is but a department,—or in common phrase, a specialty of medicine and general surgery," that medical schools must afford better facilities for preparation, and a more thorough system of training and education for such as may choose to adopt dentistry as a profession. We are told, that the course of instruction in medical schools is broader than has been adjudged needful for dental colleges, or students of dental surgery,—that the Degree, confered by medical schools, is more dignified and commanding,—that it covers and obscures the D. D. S. and that the M. D. would not only indicate higher attainments and greater capacity in our special sphere, but would abolish all distinctions between physicians and dentists, to the eminent advantage and benefit of the dentists.

Now Gentlemen of the Society of Alumni, it is no necessary for us to repeat before you, what we have so often, and so emphatically asserted before other professional organizations, or for us to challenge, as we have often done, the expression of a higher veneration or greater admiration than we have always cherished for the science of medicine, for its schools, its learning, its benevolence, its distinctive Degree and its long and illustrious line of scholars, teachers, authors and practitioners, all have our unqualified esteem and regard ; no one can no more highly appreciate or thoroughly enjoy the closest intimacy or the most cordial association with medical men, and medical schools, with medical books and medical literature, and we do not mean to reflect upon, or disparage physicians or medical institutions, when we assume that neither the one, or the other, nor both combined, are the parties or the establishment to which a student of dental surgery must apply, if he would fit and qualify himself for the present demands of society upon his profession.

The object, design, and all the provisions and arrangements of medical schools, are to develop and to teach medical service,—to make learned and skillful physicians and surgeons in the general sense. Medical schools wisely estimating their resources and justly measuring the capabilities of men, have not essayed the teaching of *universal science;* they have been content, when they have succeeded in making of their pupils and students, respectable physicians and general surgeons.

It is true, that the curriculum of the medical schools, embrace subjects of vital—of indispensable value and importance to the dentist, just as it embraces subjects of interest to the Naturalist, the Chemist, the Pharmacist and the Sculptor, but the curriculum of our own schools include the same subjects,—which are taught by medical professors —and application to the needs of dental surgery, *as they have never yet been taught in any medical school of the world.*

It is known to you all, that these medical schools have been open to the students of dental surgery for over a century, and so unsatisfactory and inadequate has been the instruction they afforded, that no encouraging result was ever realized—neither schools or students were materially benefitted, and thirty six years ago, it became a great public necessity, that better and more reliable facilities should be provided for the education of dental surgeons— that a special and distinct institution should be established for the training and preparation of a better class of practitioners than medical schools or private instructors had ever succeeded in producing, and Mr. President, *this institution, this* "*Baltimore College,*" was the "outcome," the "first born" of that imperative demand, and nobly did she respond to the claims of science, and the demands of humanity: right faithfully has she fulfilled her mission and performed her work.

She took an unlicensed, an abused and a degraded *art*, and has made of it an acknowledged and respected science; she has created and fostered a literature, which has enlarged our views, enlightened our minds, and which is daily strengthening our claims as men of science. She has sent throughout the land, and all over christendom, hundreds of well taught and skillful practitioners, some of whom, have achieved imperishable renown as operators, as authors, and as teachers. She has furnished the model, and ground work of all the schools which have subsequently been established in this country, or abroad. She is still in successful operation, dispensing her priviliges and advantages, adding yearly to her resources and facilities; and she maintains her proud pre-eminence as first of our professional institutions. But yet, in the face of this array of indisputable facts, we are told that our special schools should be abandoned; that some of our graduates and degree men do not make a favorable, or respectable impression as men of science and letters, that Alma Mater should be stricken down and a "new departure" taken. Now, sirs, it is well known that we lay under contribution, every department of medical science that relates even remotely to the exigencies and demands of dental surgery; we secure the best available talent from both professions as instructors; our course is as broad and as long, and our terms and conditions for graduation are as stringent, and exacting, as obtain in the medical or any other schools of science. Why should our students not be well and thoroughly instructed in our own colleges? and do not incontrovertible facts and results *prove*, that they *are* so instructed?

What system, from the schools of Athens down to those of the present day, could ever claim immunity or exemption from "fools and knaves?" With all my respect and veneration for medicine, her institutions, her literature and her splendid achievements, I am pained to confess, that there is hardly a community in the land, in which we may not find some shabby disciple of Æsculapius, who dishonors his Alma Mater by his stupidity and his ignorance, and who prostitutes his degree by becoming a charlatan, or a bungler. Is it fair to judge medical schools by such examples?—neither is it fair or just to so judge our own schools.

But it would be a grand thing to have our dentists accomplished physicians and general surgeons; to have them all "M. D's," so that they could on all occasions fraternize with medical men, and be recognized as their social and professional peers, and be received as acceptable members of medical associations, conventions, etc. So it would Mr. President, but for higher reasons and more creditable objects. It would be still a grander thing, if our dentists could not only be physicians, but philosophers, statesmen, orators and poets, naturalists, mathematicians, living and walking encyclopedias of all human knowledge but unfortunately, "life is short," generally, human capacity is limited; we have only a few of those wonderful intellects, of those richly endowed dental students, too few, to supply the present demand for skillful and accomplished practitioners.

The necessities of life press upon us; the few short years allotted to preparation for the duties and offices of a single profession, as a rule, tax all our energies and abilities. It is a good thing, for a dentist to be also a well taught physician, but society and common usage makes no demand upon the dentist for the offices and ministrations of the physician, and as a rule, dental students have neither the time nor the money to acquire a profession they never expect to practice, or from which they can never hope to derive a compensating return.

It has been suggested by the ablest authority we have seen upon the question we are combatting, that students of dental surgery, to be properly trained and taught, should take a *double* course of instruction, a fall and winter course in the medical, and a spring and summer course in the dental schools; to have the lecture terms so arranged, as that one would immediately succeed the other, and as we understand the suggestion, that the student should prosecute this double course to *graduation in both schools.* Now gentlemen, with all our admiration, personally and professionally for the author of the above suggestion, it strikes us that such a requirement would practically disrupt or break down all systematic training for our students; a double course of study, a double set of fees and expenses, would speedily do the business; and graduates from private offices, after a six weeks pupilage, would again begin to appear.

For students of dental surgery, observation and experience has demonstrated that our only sure reliance is upon schools of dental surgery, where every facility is provided, where every essential branch of medical science and every division and department of art is made tributary to a system of instruction, suited and adapted to the nature and requirements of what is now recognized as a distinct science, and a separate and distinct profession. "Men do not gather grapes of thorns, nor figs of thistles;" common observation, common experience and common sense, have long since determined the question, that a theological seminary is not the place to qualify a lawyer, that a military academy, is not the place to train and teach a preacher, that a school of art is not the place to make a physician, and it is equally potent, and equally true, that a medical college is not the place to make a dental surgeon.

But, say our progressive friends, our relations are so intimate, the two sciences are so kindred, that there should be no dividing line, but both should be taught and practiced under one regime. Gentlemen, all science is kindred, all departments, divisions and sub divisions, are more or less intimately related; yet all have their distinguishing characteristics and requirements, dental surgery being no exception; and surely the time has come, when this department can stand alone, and maintain something better than a parasitic existence.

Mr. President and gentlemen: In all that we have said, we meant not to underate, or disparage the value of medical teachers; it is a good, and desirable thing, for a dentist to be an accomplished and skillful physician; but we do say, that this will not, and cannot make the dentist. Dental science cannot develop, and grow, and flourish in medical schools; it has *never done so*; the experiment has been made, and repeated for a century and a half, and always with unsatisfactory and discouraging results. This, oldest of the Colleges of dental surgery, as we have said, has not reached her fourth decade; compare her thirty-seven years and their results, with all that was accomplished by the aid of medical schools in the hundred preceding years.

What was dental science forty years ago? and what is it now? And who can deny this, and other sister institutions of the same character, their founders—their co-laborers the Alumni they have sent into fields of practice all over the world, the honor and merit involved in the change?

And now in concluding this already too lengthy address, permit us to say, that in a large degree,—to the Alumni of this school is committed the responsible and important trust of *maintaining* the present status of our profession, of advancing its standard, and promoting its future growth and development

Let us not thoughtlessly or hastily surrender our "Shiholeth;" let us not be led away from the only system of professional discipline and education that has proven effective and compensative, by a desire for needless change or doubtful experiment; let us stand by and hold fast, that we have *tried* and found *true;* let us sustain by our means, our influence and example, our own institutions Let us not only give these annual expressions of moral support as an organized and working association, but let us as individual members, in our social and professional intercourse, so illustrate the lessons that we have been taught, as to honor our Alma Mater, honor ourselves, and honor and dignify the profession we represent. Let us so live, and so act, so study and so practice, as not only to retain the confidence and esteem we now enjoy, but to enhance the appreciation of society, and command the recognition and respect of all learned and liberal professions. Let us do this, and Alma Mater will be strengthened, sustained, and forever established. Let us do this, and dental science will keep step in the march of progress and improvement with all other sciences and professions; we shall command recognition as men of science: we shall be the acknowledged peers of the representatives of all the other departments of science; we shall be accorded all the social and professional consideration the most ambitious among us may covet, and our "Foster Mother," "when asked for jewels, may justly, if not proudly, point to her Sons."

